# Overcoming heterogenity in imatinib-resistant gastrointestinal stromal tumor

**DOI:** 10.18632/oncotarget.27277

**Published:** 2019-10-29

**Authors:** César Serrano, Jonathan A. Fletcher

**Affiliations:** Department of Medical Oncology, Vall d’Hebron Hospital, Barcelona 08035, Spain; Department of Pathology, Brigham and Women’s Hospital, Harvard Medical School, Boston, MA 02115, USA

**Keywords:** GIST, imatinib, sunitinib, regorafenib, therapeutic combination

Gastrointestinal stromal tumor (GIST) is the most common malignant neoplasm of mesenchymal origin. Activating mutations in KIT or PDGFRA receptor tyrosine kinases (RTKs) are central to GIST biology and drive GIST growth and progression [1]. This oncogenic addiction to KIT/PDGFRA signaling explains the profound impact of their inhibition with first-line imatinib on metastatic GIST patients’ outcomes [2]. However, disease progression eventually occurs in approximately two years after treatment initiation largely due to the polyclonal emergence of resistant subpopulations harboring different KIT secondary mutations [3]. Resistance mutations are not random and cluster in two regions of the KIT kinase domain: the ATP binding pocket (encoded by exons 13 and 14) and the activation loop (encoded by exons 17 and 18). Thus, KIT reactivation due to secondary resistance mutations in KIT underscores the prominent role of KIT as the disease driver and firmly supports therapeutic strategies aiming to suppress KIT signaling after imatinib failure. Consequently, drug development in imatinib-resistant GIST during the past decade focused on tyrosine kinase inhibitors (TKIs) targeting a broader spectrum of KIT-mutant oncoproteins, and sunitinib (second-line) and more recently regorafenib (third-line) were granted with worldwide approval [4, 5]. Of note, sunitinib and regorafenib, although effective, display a modest activity, with a median progression free survival between four to six months. Several other TKIs with KIT inhibitory activity have been or are currently being studied in phase I to phase III clinical trials showing similar efficacy irrespective of the single-agent TKI used [6].

In a recent publication [7], we demonstrated that the molecular basis for the limited clinical benefit observed with successive lines of treatment in imatinib-resistant GIST roots in their drug-specific activity profile against a subset of the KIT secondary mutational spectrum. Importantly, KIT primary genotype predicts the efficacy of imatinib in TKI-naïve GIST, and KIT secondary genotype to sunitinib. Our studies in human GIST cell lines and transfected models showed that KIT secondary genotype predicted the activity of third-line regorafenib, displaying KIT oncogenic signaling suppression among most activation-loop mutants. Specifically, regorafenib effectively inhibited, to a greater or lesser extent, resistant secondary mutations emerging in KIT codons D820, N822, Y823, several in D816 (i.e.: D816E, D816H) except for D816V, and likely A829P. *KIT* exon 14 T670I gatekeeper mutation was also suppressed by regorafenib, but not *KIT* exon 13 V654A resistance mutation, the most common secondary mutation present at the onset of imatinib failure. These results were further corroborated in biopsies from GIST patients treated with regorafenib in the phase II trial, underscoring V654A mutation as the main vulnerability for regorafenib treatment. Remarkably, we found that all TKIs, either approved or investigated in clinical trials, had activity profiles targeting only a subset of KIT secondary mutations (Figure 1). Therefore, disease progression occurs earlier after imatinib failure due to the outgrowth of cross-resistant subpopulations not suppressed by any given single-agent TKI.

**Figure 1:**
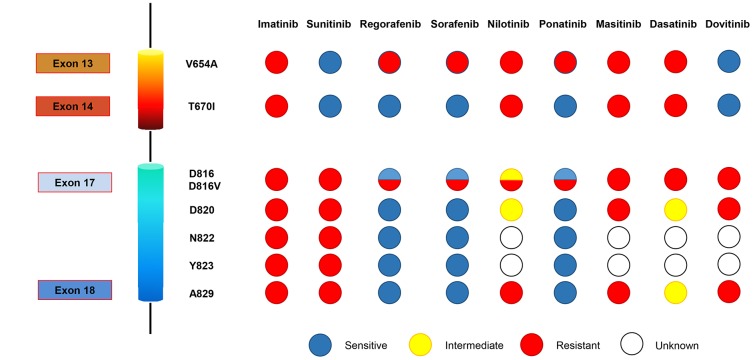
KIT secondary mutations and predicted activity profile of TKIs approved or clinical investigated for the treatment of GIST. Blue, yellow and red colors denote sensitive, intermediate activity and resistance, respectively. This panel of activity is based on our *in vitro* work [7] and on review of the published literature for these compounds.

In the absence of approved drugs with pan-KIT inhibitory activity, novel therapeutic strategies are urgently needed in order to overcome the inter- and intra-tumor heterogeneity of subclones harboring different KIT secondary mutations. Interestingly, several TKIs had complementary profiles against secondary resistant mutations, including second- and third-line approved agents for advanced GIST, sunitinib and regorafenib, respectively. Combination of these two drugs widens the spectrum of secondary-resistant clones effectively targeted and may augment the magnitude and/or duration of clinical response. However, dose reduction due to overlapping effects might preclude activity in patients. We first established co-cultures of barcoded GIST cell lines containing clinically relevant KIT primary and secondary mutations, thus representing the polyclonal imatinib-resistance heterogeneity observed in many GIST patients. We subsequently modeled preclinically a rapid alternation treatment schedule of sunitinib and regorafenib, to maintain selective pressure against polyclonal secondary-resistance GIST cells, thereby impeding the regrowth of targeted subclones when the relevant drug for those subclones is withheld. Alternation of three days of sunitinib followed by four days of regorafenib was more effective than either drug alone inhibiting cell proliferation of these polyclonal populations and preventing emergence of a single dominant clone. *In vivo* modeling of GIST polyclonality was challenging and eventually unsuccessful due to biologic differences in cell invasioin (particularly, GIST cells with KIT secondary mutations) and growth. Together, we established the rationale for a phase I clinical trial that tested, for the first time in cancer, a rapid alternation treatment-schedule using agents with complementary activity against resistant subclones (NCT02164240).

The intrinsic heterogeneity of resistant subpopulations challenges standard management of cancer patients based on the sequential use of single-agent therapies that are discontinued upon progression or unbearable toxicities. Further, although combinations of kinase inhibitors have a biologic rationale highly needed, clinical development has encountered difficulties due to enhanced and/or overlapping toxicities [8]. In this context, rapid alternation of TKIs with complementary activity against resistant clones emerges as an innovative therapeutic strategy that may allow the combinations of drugs at effective doses while maximizing treatment tolerance. Other determinants for treatment efficacy and tolerability, such as plasma drug availability and on-target activity need to be carefully assessed in the clinical trial. On the other hand, we found drug-specific activity profiles in all TKIs against specific subsets of KIT secondary mutations. This – besides explaining the limited clinical benefit of TKIs in imatinib-resistant GIST – may be an appealing opportunity to boost the clinical development of circulating tumor DNA-guided treatment of imatinib-resistant GIST in a timely and precise manner.

## References

[1] Corless CL, et al. Nat Rev Cancer. 2011; 11:865–878. 10.1038/nrc3143. 22089421

[2] Demetri GD, et al. N Engl J Med. 2002; 347:472–480. 10.1056/NEJMoa020461. 12181401

[3] Liegl B, et al. J Pathol. 2008; 216:64–74. 10.1002/path.2382. 18623623 PMC2693040

[4] Demetri GD, et al. Lancet. 2006; 368:1329–1338. 10.1016/S0140-6736(06)69446-4. 17046465

[5] Demetri GD, et al. Lancet. 2013; 381:295–302. 10.1016/S0140-6736(12)61857-1. 23177515 PMC3819942

[6] Serrano C, et al. Target Oncol. 2017; 12:277–288. 10.1007/s11523-017-0490-9. 28478525

[7] Serrano C, et al. Br J Cancer. 2019; 120:612–620. 10.1038/s41416-019-0389-6. 30792533 PMC6462042

[8] Garraway LD, et al. Cancer Discov. 2012; 2:214–226. 10.1158/2159-8290.CD-12-0012. 22585993

